# Influenza Neuraminidase Subtype N1: Immunobiological Properties and Functional Assays for Specific Antibody Response

**DOI:** 10.1371/journal.pone.0153183

**Published:** 2016-04-07

**Authors:** Don Changsom, Hatairat Lerdsamran, Witthawat Wiriyarat, Warunya Chakritbudsabong, Bunpote Siridechadilok, Jarunee Prasertsopon, Pirom Noisumdaeng, Wanibtisam Masamae, Pilaipan Puthavathana

**Affiliations:** 1 Department of Microbiology, Faculty of Medicine Siriraj Hospital, Mahidol University, Bangkok-noi, Bangkok, Thailand; 2 Center of Research and Innovation, Faculty of Medical Technology, Mahidol University, Nakhon Pathom, Thailand; 3 Department of Preclinic and Applied Animal Science, Faculty of Veterinary Science, Mahidol University, Nakhon Pathom, Thailand; 4 National Center for Genetic Engineering and Biotechnology (BIOTEC), National Science and Technology Development Agency, Pathumthani, Thailand; 5 Faculty of Public Health, Thammasat University, Pathumthani, Thailand; 6 Center for Emerging and Neglected Infectious Disease, Mahidol University, Nakhon Pathom, Thailand; University of Georgia, UNITED STATES

## Abstract

Influenza neuraminidase (NA) proteins expressed in TK^−^ cells infected with recombinant vaccinia virus carrying *NA* gene of highly pathogenic avian influenza H5N1 virus or 2009 pandemic H1N1 (H1N1pdm) virus were characterized for their biological properties, i.e., cell localization, molecular weight (MW), glycosylation and sialidase activity.

Immune sera collected from BALB/c mice immunized with these recombinant viruses were assayed for binding and functional activities of anti-NA antibodies. Recombinant NA proteins were found localized in cytoplasm and cytoplasmic membrane of the infected cells. H1N1pdm NA protein had MW at about 75 kDa while it was 55 kDa for H5N1 NA protein. Hyperglycosylation was more pronounced in H1N1pdm NA compared to H5N1 NA according to *N*-glycosidase F treatment. Three dimensional structures also predicted that H1N1 NA globular head contained 4 and that of H5N1 contained 2 potential glycosylation sites. H5N1 NA protein had higher sialidase activity than H1N1pdm NA protein as measured by both MUNANA-based assay and fetuin-based enzyme-linked lectin assay (ELLA). Plaque reduction assay demonstrated that anti-NA antibody could reduce number of plaques and plaque size through inhibiting virus release, not virus entry. Assay for neuraminidase-inhibition (NI) antibody by ELLA showed specific and cross reactivity between H5N1 NA and H1N1pdm NA protein derived from reverse genetic viruses or wild type viruses. In contrast, replication-inhibition assay in MDCK cells showed that anti-H1N1 NA antibody moderately inhibited viruses with homologous NA gene only, while anti-H5N1 NA antibody modestly inhibited the replication of viruses containing homologous *NA* gene and *NA* gene derived from H1N1pdm virus. Anti-H1N1 NA antibody showed higher titers of inhibiting virus replication than anti-H5N1 NA antibody, which are consistent with the results on reduction in plaque numbers and sizes as well as in inhibiting NA enzymatic activity. No assay showed cross reactivity with reassorted PR8 (H1N1) virus and H3N2 wild type viruses.

## Introduction

Influenza virus contains two major glycoprotein spikes: neuraminidase (NA) and hemagglutinin (HA) on the virion surface. HA binds sialic acid receptor on the host cell membrane and serves as the major target for neutralizing antibodies [1], whereas NA cleaves terminal sialic acid residues promoting the release of progeny virions [2]. NA may also contribute to the initial step of viral infection by promoting virus entry [3] or removing decoy receptors from human airway epithelial cells [4, 5], facilitating virus invasion.

Virions contain 4–5 times more HA molecules than NA molecules; thus, HA is the immunodominant antigen for the induction of antibody responses [6]. It has been suggested that anti-HA exhibits complete protection, while anti-NA exhibits partial protection [7–10]. Epidemiological data in a human population suggested that pre-existing antibody to N2 NA induced by prior infections with the pandemic Asian influenza A (H2N2) virus in 1957 might have contributed to the partial protection against the pandemic caused by Hong Kong A (H3N2) virus in 1968 [7, 8]. Subsequently, several studies employed animal models to explore the role of NA immunity against influenza virus. It was demonstrated that NA immunity conferred partial protection, or at least exhibited a less severe clinical outcome that was correlated to the level of NA antibody [9–13]. Various sources of NA proteins have been used for animal immunization to study NA immunity, e.g., DNA vaccines [9, 11], yeast expression system [14, 15], recombinant vaccinia virus [16–18] or modified vaccinia virus Ankara strain [19], baculovirus-insect cell system [20–23] and virus-like particles (VLP) [24, 25]. Nevertheless, the NA proteins produced in those studies were not well characterized; and the methods to determine functional activities of NA antibody against homologous and heterologous NA strains and subtype are quite limited.

HA antibody at titers equal to or greater than 40 as measured by hemagglutination-inhibition (HI) assay were suggestive of 50% protection against influenza virus infection [26]. Moreover, microneutralization (microNT) assay is employed as a method to measure protective antibody [26, 27]. However, there is no standard *in vitro* assay to measure protection mediated by anti-NA antibody. Most of the studies measured NA antibodies using enzyme-linked immunosorbent assay (ELISA) that determines the binding activity of anti-NA antibody, or by NA-inhibition (NI) assays that measure the antibody which blocks NA enzymatic activity employing thiobarbituric acid (TBA)-based assay or fetuin-based enzyme-linked lectin assay (ELLA) [9, 28–30]. Few studies have directly demonstrated the function of anti-NA antibody on the inhibition of virus infection/replication. These studies were based on the reduction in number of plaques or plaque size or blocking virus exit from host cells [12, 31–33].

In the present study, recombinant vaccinia viruses harboring *NA* gene of highly pathogenic avian influenza (HPAI) H5N1 or 2009 pandemic H1N1 virus were constructed. The recombinant NA proteins expressed in the infected thymidine kinase negative (TK^−^) cells were characterized in comparison to those produced in the Madin-Darby canine kidney (MDCK) cells infected by the parental wild type viruses. Antisera collected from mice immunized with these recombinant viruses were assayed for binding and functional activities of anti-NA antibodies against homologous and heterologous strains and subtypes using ELLA, plaque reduction assay and replication-inhibition assay which mimics microneutralization test.

## Material and Methods

### Wild Type Influenza Viruses

Influenza viruses used in this study were two HPAI H5N1 viruses including A/Thailand/1(KAN-1)/2004 clade 1 virus (KAN-1 virus) and A/Laos/Nong Khai 1/2007 clade 2.3.4 virus (NK-1 virus); four of H1N1 pandemic 2009 viruses including A/Thailand/104/2009 (H1N1) virus (TH 104 virus-a strain isolated from the second imported case in Thailand), A/Thailand/SEA-001(34)/2010 (H1N1) virus (a H1N1pdm virus isolated from the 2nd epidemic wave), A/Thailand/RMSC_18/2010 (H1N1) virus (a H1N1pdm virus from the 3rd epidemic wave), and A/Thailand/NMA_1/2014 (H1N1) (a H1N1dpm virus isolated in 2014). Two H3N2 strains including A/Thailand/NKS_1/2011 (H3N2) (A/Perth/16/2009-like/virus) and a local isolate A/Victoria/361/2011-like virus were included as controls. These viruses were propagated in MDCK cells maintained in Earle’s minimal essential medium (EMEM) (Gibco, Life Technologies, Grand Island, NY) without serum supplement. The virus growth media, except for that of HPAI H5N1 virus, was also supplemented with trypsin-tosylphenylalanylchloromethyl ketone (trypsin-TPCK) (Sigma-Aldrich, St. Louis, MO).

Vaccinia virus strain Lister was kindly provided by the Thai Government Pharmaceutical Organization. This vaccinia vaccine strain was used as the parental virus for the construction of recombinant vaccinia viruses carrying *NA* gene of H5N1 KAN-1 (rVac-H5N1 NA virus) or TH 104 virus (rVac-H1N1 NA virus). Both the vaccinia vaccine strain and the recombinant viruses were propagated in TK^−^ cells and maintained in EMEM supplemented with 2% fetal bovine serum (FBS) (Gibco).

### Recombinant Vaccinia Viruses

The rVac-H1N1 NA virus was constructed as described previously for rVac-H5N1 NA virus [34]. Briefly, total RNA was extracted from MDCK cells infected with TH 104 virus using a QIAamp viral RNA mini kit (Qiagen, Venlo, Netherlands). The complete *NA* gene was amplified by a OneStep RT-PCR kit (Qiagen) using universal primers designed by Hoffmann, et al [35] but modified in this study by overhanging the 5′ end of each primer with a restriction site for the enzyme *Xma*I. The sequences of this primer pair were SXm-NA-1: 5′-TATTCCCGGGAGAGCAAAAGCAGGAGT-3′ and SXm-NA-1413R: 5′-ATATCCCGGGTATTAGTAGAAACAAGGAGTTTTTT-3′. The amplified products were cloned into a pGEM-T Easy vector using T4 DNA ligase (Promega Corporation, Madison, WI) before being transformed into *Escherichia coli* JM109. Thereafter, the recombinant plasmids were digested with *Xma*I (New England Biolabs Inc., Ipswich, MA) and subcloned into the pSC11 expression vector (kindly provided by Prof. Bernard Moss, the National Institute of Allergy and Infectious Disease, Maryland, USA) which was also cut by *Xma*I. The insertion site of *Xma*I is located downstream of the vaccinia virus p7.5 promoter together with the *E*. *coli lacZ* gene that encodes for β-galactosidase under a p11 promoter, and is flanked with thymidine kinase sequences (TK_R_ and TK_L_) [36]. *E*. *coli* was transformed with the recombinant pSC11 plasmids and plated onto Luria-Bertani agar containing 5-bromo-4-chloro-3-indolyl-β-D-galactopyranoside (X-gal) (Promega) plus ampicillin as a selective marker. The recombinant plasmids were extracted, checked for orientation of the NA DNA insert by cutting the purified recombinant plasmids with restriction enzymes *Bam*HI (New England Biolabs) and the sequence of the insert gene was confirmed by DNA sequencing.

To construct rVac-H1N1 NA virus, a mixture of pSC11 recombinant plasmids and DMRIE-C transfection reagent (Invitrogen, Eugene, OR) was inoculated onto the TK^−^ cell monolayer pre-infected with wild type vaccinia virus at a multiplicity of infection (m.o.i.) of 0.01 plaque forming unit (pfu)/ml for 2 hours. The transfected culture was further incubated for 2 days to allow virus replication. The recombinant vaccinia virus was differentiated from the wild type vaccinia virus by plaque selection on the TK^−^ cell monolayer maintained in low melting point agarose containing 5-bromo-2′-deoxyuridine (Sigma Aldrich) and X-gal, in which the plaques produced by cells infected with the recombinant vaccinia virus appeared blue. Plaque purification was performed three times to obtain a single clone of the recombinant virus that was further propagated and titrated in TK^−^ cells by plaque assay.

### Reverse Genetic Influenza Viruses

Reassortant viruses containing *HA* and/or *N*A genes of interest were constructed by reverse genetic technique using 8 recombinant pHW-2000 plasmid backbones of A/Puerto Rico/8/1934 (H1N1) (PR8 virus) origin [37]. This set of reverse genetic plasmids was kindly provided by Prof. Robert G. Webster, St. Jude Children Research Hospital, Memphis, TN. Briefly, complete *HA* and *NA* genes were amplified using universal primers [33]. The amplification products were cloned into pGEM-T easy vector (Promega) and subcloned into pHW-2000 plasmids. The cleavage site in the HA protein was modified from PQRERRRKKR of the HPAI H5N1 virus to PQ—-IETR, the amino acid sequence of avirulent H6 virus, to eliminate the virulence of the constructed reverse genetic (rg) virus. Subsequently, the recombinant plasmids in combination with the other 6 or 7 recombinant plasmids carrying the internal genes from PR8 virus in *Trans*IT-LT1 solution (MirusBio, Madison, WI) were used to transfect the co-cultures of MDCK cells and human embryonic kidney 293 T (HEK-293T) cells maintained in Opti-MEM (Gibco). The inoculated cell monolayers were incubated at 37°C in a CO_2_ incubator and observed daily for cytopathic effects (CPE).

Four rg-viruses were generated in the present study, including the rgPR8 parental virus, rgH5N1-HANA (6+2) and rgH5N1-NA (7+1) clade 2.3.4 virus derived from NK-1 (H5N1) virus, rgH1N1-HANA (6+2) and rgH1N1-NA (7+1) viruses derived from TH 104 H1N1pdm virus. Moreover, this study also included two rg-viruses, i.e., rgH5N1-HANA (6+2) and rgH5N1-NA (7+1) clade 1 viruses derived from KAN-1 (H5N1) virus kindly provided by Prof. Prasert Auewarakul, Mahidol University, Thailand.

### Immunofluorescence Assay

Confocal immunofluorescence assay (IFA) was conducted to demonstrate the expression and localization of NA proteins in TK^−^ cells infected with recombinant vaccinia viruses. The cells infected with rVac-H1N1 NA virus were stained with goat antiserum against N1 NA kindly provided by Prof. Robert G. Webster; and cells infected with rVac-H5N1 NA virus were stained with goat antiserum against recombinant NA proteins from A/Vietnam/1203/2004 (H5N1) and A/Hong Kong/483/1997 (H5N1) viruses kindly provided by BEI Resources through the NIH Biodefense and Emerging Infections Research Resources Repository, NIAID, NIH (catalog number NR-9598). Fluorescein isothiocyanate (FITC) conjugated-rabbit anti-goat Ig (Dako Cytomation, Glostrup, Denmark) was used as the secondary antibody. The slides were then counter stained with trihydrochloride trihydrate (Hoechst 33342-Invitrogen, Eugene, OR) and examined for presence of the recombinant NA protein under a laser scanning confocal microscope (LSM 510 Meta, Zeiss, Jena, Germany).

Conventional IFA was also performed to determine the titers of anti-NA binding antibodies in mouse sera collected at 4 or 6 weeks after immunization. Deposits of MDCK cells infected with the wild type virus were used as the test antigen, and FITC-conjugated goat anti-mouse Ig (SouthernBiotech, Birmingham, AL) was used as the secondary antibody. The reciprocal of the highest serum dilution that yielded 2+ fluorescence intensity was consider the antibody titer.

### Western Blot Assay

Western blot (WB) assay was performed to determine the molecular weight (MW) of NA expressed in TK^−^ cells infected with recombinant vaccinia virus or MDCK cells infected with wild type influenza virus. The infected cell pellets were lysed with RIPA buffer containing 50 mM TrisCl pH 7.5, 150 mM NaCl, 1% Triton X-100, 0.5% sodium deoxycholate, and 0.1% sodium dodecyl sulphate (SDS). Reference NA proteins included recombinant NA derived from A/Vietnam/1203/2004 (H5N1) virus expressed in mouse myeloma cells (NS0) (r-H5N1 NA) (R&D Systems, Minneapolis, MN) and recombinant NA protein derived from A/California/04/2009 (H1N1) virus expressed in the baculovirus-insect cell system (r-H1N1 NA) (kindly provided by BEI Resources, catalog number NR-19234). Cell lysates or reference antigens were mixed with 4× reducing sample buffer (8% SDS, 250 mM Tris Cl pH 6.8, 8% β-mercaptoethanol, 0.4% bromophenol blue, 40% glycerol) and boiled for 10 minutes prior to electrophoresing in 12% denaturing discontinuous sodium dodecyl sulphate-polyacrylamide gel electrophoresis (SDS-PAGE). The electrophoresed proteins in gel were then blotted onto a nitrocellulose membrane (Pall, Port Washington, NY) using Trans-Blot semidry transfer cell (Bio-Rad, Hercules, CA). The blotted membrane was blocked with 1% bovine serum albumin in phosphate buffer saline plus 0.1% Tween-20 and incubated with rabbit polyclonal antibody against NA protein of A/Vietnam/1203/2004 (H5N1) or rabbit polyclonal antibody against NA protein of A/California/06/2009 (H1N1) (eEnzyme, Gaithersburg, MD) Thereafter, the membrane was incubated with horseradish peroxidase (HRP) conjugated-goat anti-rabbit IgG (Perkin Elmer, Waltham, MA) as the secondary antibody and a mixture of 3, 3′diaminobenzidine (Sigma-Aldrich), 8% NiCl_2_, and H_2_O_2_ was used as the chromogenic substrate.

### Deglycosylation of NA Proteins

Recombinant NA proteins expressed in TK^−^ cells infected with rVac-H5N1 NA or rVAc-H1N1 NA virus were investigated for post-translational glycosylation in comparison to those synthesized in MDCK cells infected with the parental wild type virus. The infected cells were lysed with RIPA followed by treatment with *N*-glycosidase F (PNGase F) (New England Biolabs) for digestion of the glycosylated protein. According to the manufacturer’s protocol, the cell lysates were mixed with 10× glycoprotein denaturing buffer and heat at 95°C for 10 minutes. The denatured proteins were then added with 10× G7 reaction buffer, 10% NP40 and PNGase F enzyme. The reaction was incubated at 37°C for 1 hour, and the digested products were visualized by WB assay.

### Neuraminidase Activity Assay

Neuraminidase (sialidase) enzymatic activity of recombinant NA proteins expressed in TK^−^ cells infected with rVac-H5N1 NA or rVAc-H1N1 NA virus was determined by MUNANA assay and ELLA. MUNANA assay was used to demonstrate the cleavage of a fluorescent substrate, 2′-(4-methylumbelliferyl)-α-D-N-acetylneuraminic acid sodium salt hydrate (MUNANA), by influenza NA; and the yield of free 4-methylumbelliferone (4-MU) fluorescent product was then quantitated based on its fluorescence intensity. Briefly, TK^−^ cells were infected with rVac-H5N1 or rVac-H1N1 NA virus at m.o.i. of 0.01 and incubated at 37°C for 3 days, the time at which the degree of the CPE was 4+. The supernatants were discarded; the virus infected cells were resuspended with 2% FBS EMEM and underwent 3 cycles of freezing and thawing to release NA proteins and viruses from the infected cells. The amounts of NA proteins expressed in TK^−^ cells infected with rVac-H5N1 NA or rVac-H1N1 NA viruses were verified by determining for the intensity of various proteins present in the infected cell lysates using SDS-PAGE and Coomassie blue staining. Furthermore, the cell lysates were also titrated for concentration of the recombinant vaccinia viruses. The cell lysates containing 5×10^8^ pfu/ml was serially diluted in a 10-folded dilution manner with 2-morpholinoethanesulfonic acid (MES) assay buffer (32.5 mM MES, 4mM CaCl_2_) and a 20 μl volume was added to 30 μl of MUNANA substrate at concentration of 100 μM. The reaction well was incubated at 37°C for 60 minutes with shaking in the dark before terminating with 150 μl of the stop solution (0.1 M glycine in 25% ethanol, pH 10.7). The reaction plate was read under a spectrophotometer to determine for relative fluorescence units (RFU) using an excitation wavelength of 355 nm and an emission wavelength of 460 nm. The RFU values were plotted against numbers of pfu to establish the dose response curve indicating the NA enzymatic activity.

The same preparation of cell lysate as described above was also investigated for NA enzymatic activity by fetuin-based ELLA using the protocol established by Dr. Maryna Eichelberger [38]. The cell lysate containing recombinant vaccinia viruses at the concentration of 2×10^8^ pfu/ml was serially 10 fold-diluted and each dilution in a 50 μl volume was added into duplicate wells of a 96 well plate pre-coated with fetuin. The recombinant NA cleaved the sialic acid side chain of fetuin and yielded small cleavage products that were washed out while leaving the carbohydrate moieties adhered to the wells. These moieties were subsequently detected by HRP conjugated-peanut lectin using *o*-phenylenediamine dihydrochloride (OPD) as the chromogenic substrate. The reaction plate was read under a spectrophotometer at an optical density (OD) of 492 nm. The OD values were plotted against numbers of pfu to establish the dose response curve indicating the NA enzymatic activity.

### Mouse Immunization

BALB/c mice were purchased from the National Laboratory Animal Center, Mahidol University and housed in the Faculty of Veterinary Science, Mahidol University. Use of experimental animals was approved by the Animal Care and Use Committee of Faculty of Veterinary Science, Mahidol University (Permit Number: MUVS-2013-37). All activities concerned with the animals were carried out by the experienced veterinarians. The physical condition of animals was monitored once a day throughout the end of the experiments, and all animals were healthy. No animal was ill or died prior to the experimental endpoint. At time of blood collection, the animals were anesthetized using isoflurane inhalation. When the animals were unconscious, whole blood was collected by cardiac puncture. Death was confirmed by physical examination. Mice of age six weeks were divided into five groups; and each group comprising 5–6 mice was inoculated intraperitoneally once with rVac-H1N1 NA, rVac-H5N1 NA, rVac-pSC11or wild type vaccinia virus plus 10 μg of poly I:C (Sigma-Aldrich) at the inoculums dose of 10^7^ pfu/head and the last group was the unimmunized control. Three independent lots of mice were used in the experiments. The mice were bled by cardiac puncture; the first lot was sacrificed at 4 weeks and the second and the third lots at 6 weeks after immunization. Mouse sera were aliquoted and stored at −20°C and pooled for anti-NA antibody testing.

### Enzyme-Linked Lectin Assay

Mouse sera were investigated for anti-NA antibody that inhibited NA enzymatic activity by ELLA. The test virus was two-fold serially diluted to titrate for NA activity before subjecting to anti-NA antibody detection as described above. Each virus dilution was plotted against its OD value to establish a titration curve. An OD value of about 2.0 was extrapolated against the titration curve to determine the optimal virus dilution for further use in neuraminidase inhibition (NI) assay.

For NI antibody assay, heat-inactivated mouse serum sample at a dilution of 1:10 was 2-fold serially diluted and added in duplicate into a fetuin coated plate together with the test virus at working concentration. After overnight incubation, the amount of carbohydrate moieties produced after digestion of sialic acid was determined as described above. The NI antibody titer was defined as the highest serum dilution that yielded a 50% reduction of the OD value compared with the virus control without serum.

### Plaque Reduction Assay

The mouse sera were treated with receptor-destroying enzyme (RDE) (Denka Seiken, Tokyo, Japan) at 37°C for 16–18 hours followed by heat inactivation at 56°C for 30 minutes. Confluent MDCK cell monolayers in 6-well tissue culture plates were infected with either TH 104, rgH5N1-HANA (6+2) clade 1 or rgPR8 virus at the inoculums dose of 50 pfu per well. The viruses were absorbed for 2 hours at 37°C and the cell monolayers were washed twice and maintained with 3 ml of 1.2% Avicel in EMEM containing RDE-treated antisera at various dilutions. The infected cell monolayers maintained with 1.2% Avicel containing either normal mouse sera or none were assayed in parallel as controls. After incubation for 48 hours at 37°C, the cell monolayers were fixed with 10% paraformaldehyde, stained with 1% crystal violet and count for number of plaques.

### Replication-Inhibition Assay

Replication-inhibition assay mimicking the microNT assay in MDCK cell monolayers [39] was established in this study. Briefly, the test sera were treated with RDE at 37°C for 16–18 hours followed by heat inactivation at 56°C for 30 minutes. The treated sera starting from a dilution of 1:20 were 2-fold serially diluted, added to the test virus at final concentrations of 100, 25 or 10 TCID50/reaction well in duplicate, and further incubated at 37°C for 2 hours. The virus-serum mixtures were transferred onto MDCK cell monolayers and incubated for 3 days. The CPE was examined daily; and at the end point measurement, CPE was scored in conjunction with testing the culture supernatant from each well for hemagglutinating activity using 0.5% goose red blood cells to determine virus release. Titer of the NI antibody was defined as the reciprocal of the highest serum dilution that showed 2+ hemagglutination pattern compared with the virus control.

## Results

### Cellular Localization of NA Proteins

**Fig 1:**
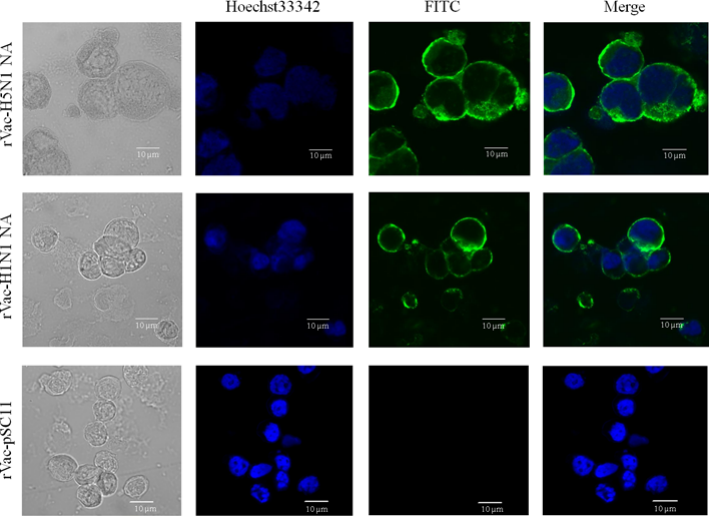
Cellular localization of NA proteins. The NA protein expressed in TK^−^ cells infected with rVac-H5N1 NA or rVac-H1N1 NA are present in cytoplasm, and in particular, on the cytoplasmic membrane as demonstrated by IFA confocal microscopy. TK^−^ cells infected with rVac-pSC11 as the virus control did not have a fluorescent signal.

Expression and localization of NA protein produced in TK^−^ cells infected with rVac-H5N1 NA or rVac-H1N1 NA virus was determined by confocal IFA. The result showed a high level of expression of recombinant NA proteins which localized both in the cytoplasm and on the cytoplasmic membrane of the infected cells (Fig 1). TK^−^ cells infected with rVac-pSC11 as the virus control did not have a fluorescent signal.

### Molecular Weights of NA Proteins

**Fig 2:**
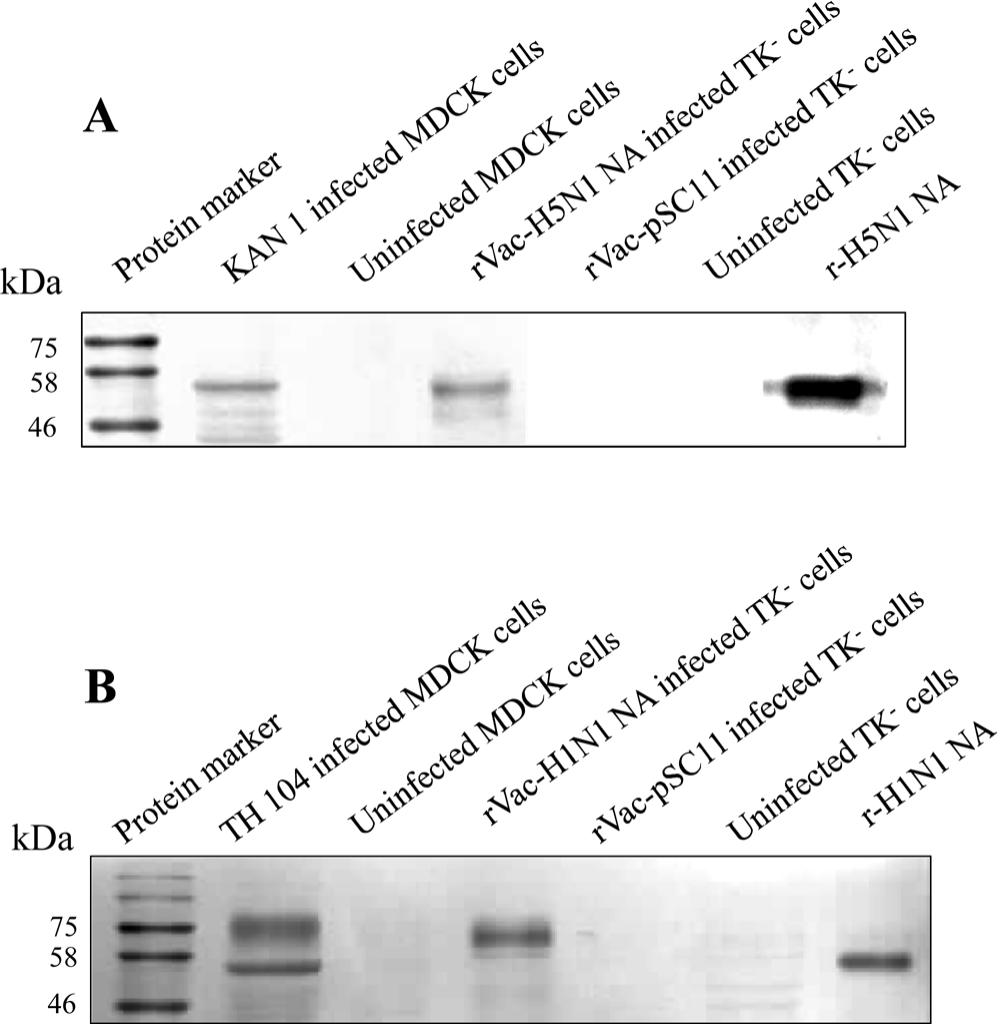
Molecular weights of NA proteins as demonstrated by WB assay. (A) The MW of rVac-H5N1 NA is compared with those NA synthesized in MDCK cells infected with wild type KAN-1 virus and the commercial recombinant H5N1 NA produced in mouse myeloma cells (NS0) (r-H5N1 NA). (B) The MW of rVac-H1N1 NA is compared with those NA synthesized in MDCK cells infected with wild type TH 104 virus and recombinant NA of A/California/04/2009 (H1N1) produced in insect cells (r-H1N1 NA).

WB assay was performed to determine the MW of NA proteins expressed in various virus-cell systems, i.e., TK^−^ cells infected with recombinant vaccinia viruses and MDCK cells infected with parental wild-type viruses. NA proteins obtained from the other sources were used as the references. TK^−^ cells infected with rVac-H5N1 NA virus or MDCK cells infected with KAN-1 H5N1 wild type virus produced NA proteins with MWs of about 55 kDa. Similarly, recombinant H5N1 NA protein produced in a mouse myeloma cell line purchased from R&D Systems also has a MW of about 55 kDa (Fig 2A). On the other hand, there was only one form of NA protein with a MW of about 75 kDa produced in TK^−^ cells infected with rVac-H1N1 NA virus; while there were two forms of NA protein at MW of 75 and 55 kDa produced in MDCK cells infected with TH 104 wild type virus. There was one form of recombinant H1N1 NA protein with a MW of about 55 kDa produced in baculovirus-insect cell system obtained from BEI Resources (Fig 2B).

### Glycosylation of NA Proteins

**Fig 3:**
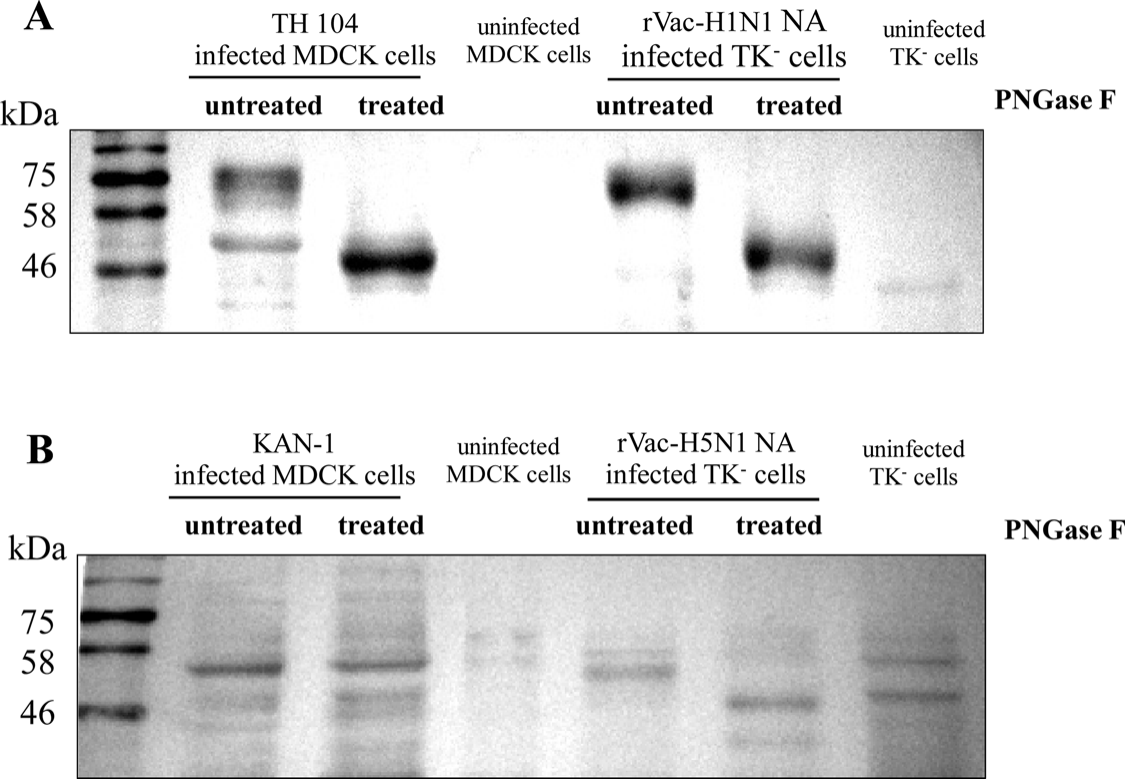
Glycosylation of NA proteins. MWs of the cleavage products of NA protein after treatment with Peptide *N*-Glycosidase F (PNGase F) in comparison with the size of the untreated NA as demonstrated by WB assay. (A) H1N1pdm NA; (B) H5N1 NA.

The differences in the patterns of H1N1 NA proteins produced from various sources suggested that their levels of post-translational glycosylation might be different. Lysates of MDCK cells infected with wild type TH 104 virus and TK^−^ cells infected with rVac-H1N1 NA virus were treated with PNGase F for deglycosylation. The result showed that the MWs of 75 and 55 kDa of H1N1 NA proteins produced in MDCK cells infected with wild type virus and that of 75 kDa produced in TK^−^ cells infected with the recombinant vaccinia virus were reduced to one form of NA protein at MW of approximately 50 kDa (Fig 3A), close to 51.6 kDa, the size of native form of H1N1 NA as predicted by using two web-based tools: the Compute pI/Mw tool (http://web.expasy.org/compute_pi/) and the protein molecular weight calculator (http://www.sciencegateway.org/tools/proteinmw.htm).

A single band of H5N1 NA at MW of 55 kDa was shown in both MDCK and TK^−^ cells. After PNGase F treatment, the MW of H5N1 NA protein produced in TK^−^ cells infected with recombinant vaccinia virus was reduced from 55 to 50 kDa, while no change in MW was observed with that produced in MDCK cells infected with wild type virus (Fig 3B).

Collectively, we showed that H1N1 NA expressed in either MDCK or TK^−^cells were hyperglycosylated by *N*-linked glycans which could be removed by PNGase F treatment. Similar finding was demonstrated in H5N1 NA expressed in TK^−^ cells infected with recombinant virus. On the other hand, our result suggested that H5N1 NA produced in the infected MDCK cells might be glycosylated by the other kind of glycosides which could not be removed by PNGase F digestion. Thus, we showed that glycosylation depends on either the host cell type or the virus strain.

### The Potential Glycosylation Sites on the NA Structures

**Fig 4:**
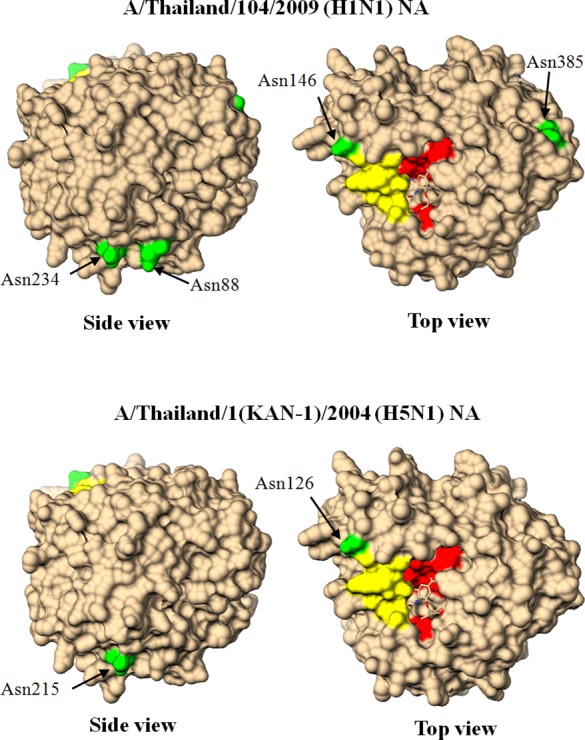
Potential *N*-linked glycosylation sites on NA globular domain. The glycosylation sites on the molecular surfaces of NA globular head domain monomer as demonstrated by three-dimensional structures based on A/Vietnam/1203/04 (H5N1) (PDB codes: 2HU0). Potential *N*-linked glycosylation sites are highlighted in green, structure of the 150-loop is highlighted in yellow and amino acids related to the NA active sites are highlighted in red.

Our previous work predicted that NA of H1N1 virus harbored 8 glycosylation sites and 4 of them were located on the NA globular head, while NA of H5N1 viruses contained only 2 sites [40]. The potential *N*-linked glycosylation motifs are defined as Asn-X-Ser/Thr, where X represents any amino acid except proline [41]. The potential glycosylation sites of KAN-1 and TH 104 NAs were mapped onto reference structure of A/Vietnam/1203/04 (H5N1) NA (PDB codes: 2HU0) using Chimera program (version 1.10.1) [42]. The globular head domain of NA protein starts from position 83 to 469 (N1 numbering). The amino acid positions that are the potential *N*-linked glycosylation sites on globular head of TH 104 NA include Asn88, Asn146, Asn235 and Asn385; and KAN-1 NA include Asn126, Asn215 (Fig 4).

### Neuraminidase Enzymatic Activity of NA Proteins

**Fig 5:**
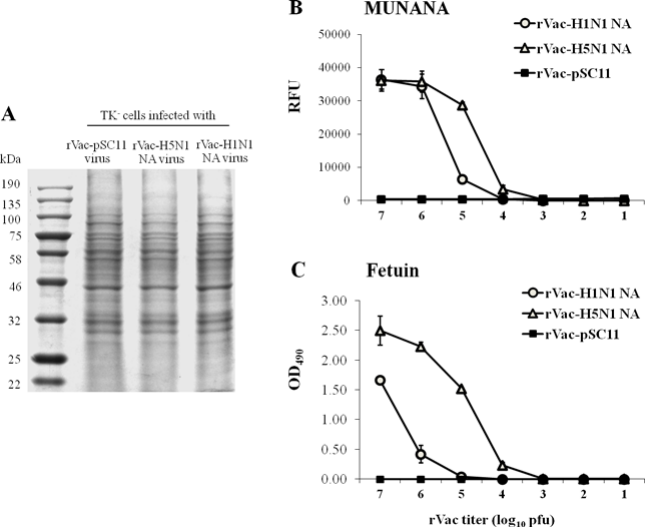
Neuraminidase enzymatic activity of NA proteins in lysates of TK^−^ cells infected with recombinant vaccinia virus harboring H1N1 *NA* gene (rVac-H1N1 NA) or H5N1 *NA* gene (rVac-H5N1 NA). (A) SDS-PAGE and Coomassie blue staining demonstrate the comparable intensity of various protein bands (viral NA and cellular proteins) in TK^−^ cells infected with rVac-pSC11, rVac-H5N1 NA or rVac-H1N1 NA virus. (B) Neuraminidase enzymatic activity of NA proteins in lysates of TK^−^ cells infected with rVac-H1N1 NA or rVac-H5N1 NA virus as determined by MUNANA-based assay; and (C) fetuin-based ELLA. The cleavage product of MUNANA substrate is measured in term of RFU; and that of fetuin substrate is measured as the OD values. The H5N1 NA protein is statistically higher in sialidase activity than H1N1 NA protein (ANOVA, *p* < 0.001). Enzymatic activity is not found in lysate of TK^−^ cells infected with rVac-pSC11 virus.

Lysates of TK^-^ cells infected with the recombinant vaccinia virus harboring H5N1 NA or H1N1 NA virus were subjected to SDS-PAGE and Coomassie blue staining to demonstrate the expression level of the viral NA. The intensity of various protein bands (viral NA and cellular proteins) derived from any of the two infected cultures was comparable, demonstrating that the H5N1 NA and the H1N1 NA were expressed at similar level (Fig 5A). Moreover, both kinds of cell lysates contained the recombinant vaccinia virus at titers of approximately 10^9^ pfu/ml.

The cell lysates were further determined for enzymatic activity of the NA sialidase by MUNANA-based assay and fetuin-based ELLA. The two methods demonstrated the enzymatic activity of H5N1 NA and H1N1 NA in a dose response curve. Nevertheless, enzymatic activity of H5N1 NA was statistically higher than H1N1 NA as determined by either MUNANA (Fig 5B) or fetuin substrate (Fig 5C) (ANOVA, *p* < 0.001 for both assays). Lysate of cells infected by rVac-pSC11 virus as negative control did not exhibit NA enzymatic activity (Fig 5B and 5C).

### NA Binding Antibodies

Pooled sera from each lot of the immunized mice were determined for NA binding antibody titer by IF assay using MDCK cells infected with wild type KAN-1 virus or TH 104 virus as the test antigens. The result showed that all 3 lots of pooled sera from mice immunized with rVac-H5N1 NA virus had antibody titer of 40, and those from mice immunized with rVac-H1N1 NA virus had the titer of 80.

### Inhibitory Effect of Anti-NA Antisera on the NA Enzymatic Activity

**Fig 6:**
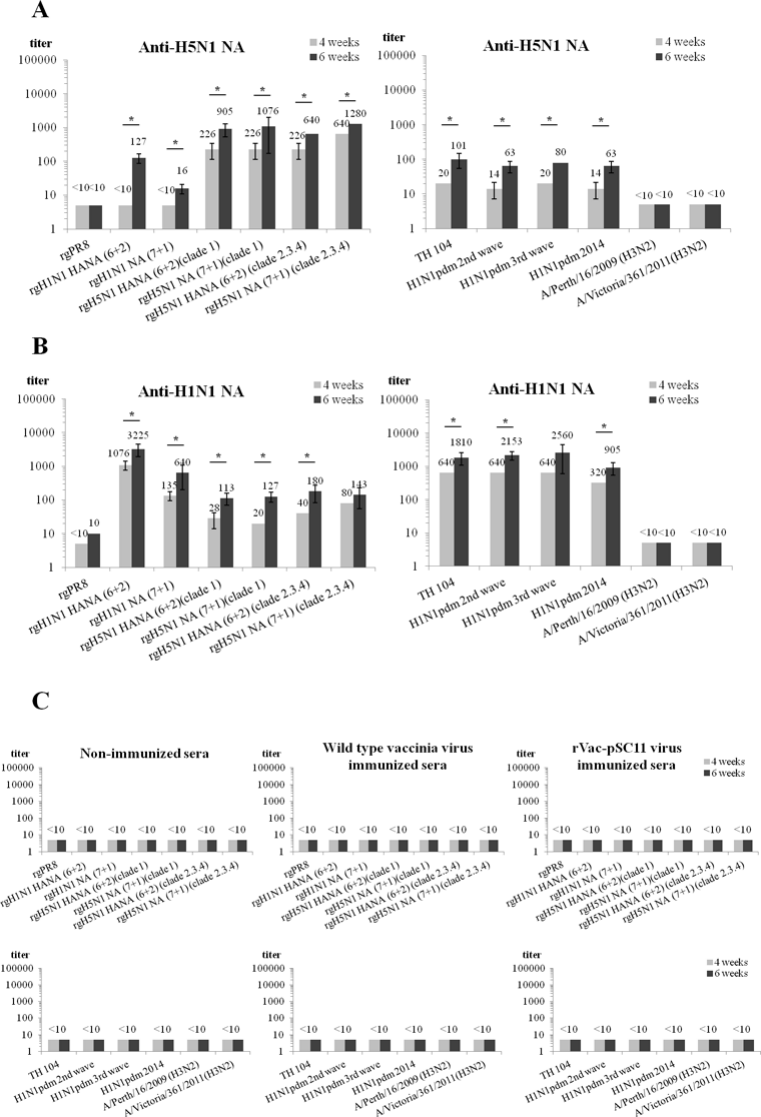
NI antibodies in sera of mice immunized with recombinant vaccinia virus harboring H5N1 *NA* gene (anti-H5N1 NA) or H1N1 *NA* gene (anti-H1N1 NA) as assayed against reverse genetic (rg) viruses (left column) and wild type influenza viruses (right column) determined by ELLA. (A) Anti-H5N1 NA antisera react with rg viruses containing homologous clade 1 *NA* gene and cross react with rg viruses containing clade 2.3.4 NA and H1N1 wild type viruses from various epidemic waves. No cross reaction is observed with rgPR8 (H1N1) virus and H3N2 wild type viruses. (B) Similar results are obtained with anti-H1N1 NA antisera. Mouse immune sera collected at 6 weeks p.i. showed significantly higher NI antibody titers than those collected at 4 weeks p.i. for the tested viruses with asterisk (Mann-Whitney U test, *p* < 0.05). (C) Normal mouse sera, sera from mice immunized with wild type vaccinia virus or rVac-pSC11 virus did not exhibit NI antibody activity. One lot of pooled sera collected at 4 weeks p.i., and 2 lots collected at 6 weeks p.i. are employed in the assay. The geometric mean antibody titers are calculated. The error bars indicate standard deviations of 2 independent experiments for each lot of the test sera.

**Fig 7:**
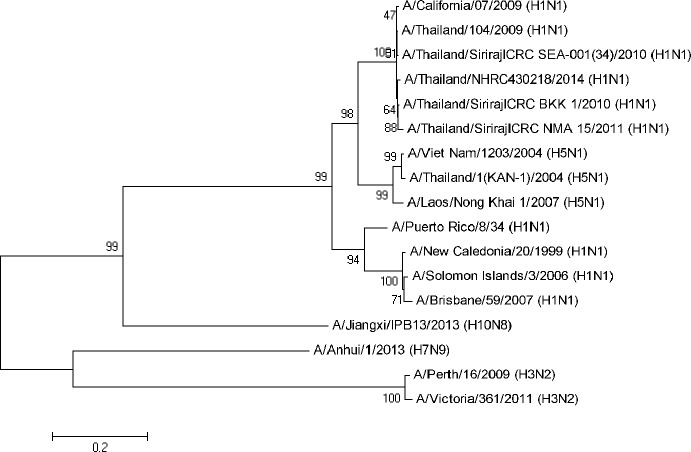
Phylogenetic tree of influenza virus *NA* constructed by neighbor-joining using MEGA 5 software. NA amino acid sequences of the H1N1pdm viruses and H5N1 viruses share the same node of origin, while H3N2 viruses are clustered in distinct node.

Immune sera from mice immunized with rVac-H5N1 NA inhibited NA enzymatic activity of both rgH5N1 clade 1 and clade 2.3.4 viruses. The antisera cross inhibited the rgH1N1 viruses, but to a lesser extent. Moreover, these mouse antisera to H5N1 NA yielded comparable NI antibody titers when H1N1 wild type viruses originating from 4 different epidemic waves were used as sources of the test NA (Fig 6A). Similarly, mouse antisera against rVac-H1N1 NA inhibited the NA enzymatic activity of rgH1N1 viruses as well as H1N1 wild type viruses, and lower NI antibody titers were obtained when rgH5N1 viruses were employed as the test NA (Fig 6B). Mouse immune sera collected at 6 weeks post immunization (p.i.) showed significantly higher NI antibody titers against almost all of the test viruses compared to the sera collected at 4 weeks p.i. (Mann-Whitney U test, *p* < 0.05) (Fig 6A and 6B). It was noted that mouse antisera against rVac-H5N1 NA or rVac-H1N1 NA virus reacted poorly with rgPR8 (H1N1) virus, and did not react with the 2 strains of H3N2 wild type viruses (Fig 6A and 6B). Normal mouse sera, sera from mice immunized with wild type vaccinia virus or rVac-pSC11 virus did not exhibit NI antibody activity (Fig 6C). Phylogenetic analysis demonstrated that KAN-1 NA and TH 104 NA belonged to the same cluster of avian origin, while PR8 NA belonged to a separate group (Fig 7). Concordant results were obtained from the two lots of mouse sera collected at 6 weeks p.i.

### Inhibitory Effect of Anti-NA Antisera on Plaque Formation

The plaque reduction assay was performed to demonstrate the role of anti-NA antibodies at virus entry or virus exit. The rgH5N1 (6+2) clade 1, TH 104 or rgPR8 viruses were used as the test viruses. To determine the inhibition of virus entry mediated by anti-NA antibodies, the test viruses were incubated with the diluted mouse antisera before inoculating onto wells of MDCK cell monolayers. The serum-virus mixtures were washed out and the infected cultures were maintained in media without anti-NA antisera. The result shows no reduction in number of plaques and plaque size mediated by anti-H5N1 NA or anti-H1N1 NA antisera, suggesting that anti-NA antibody did not inhibit the virus entry.

**Fig 8:**
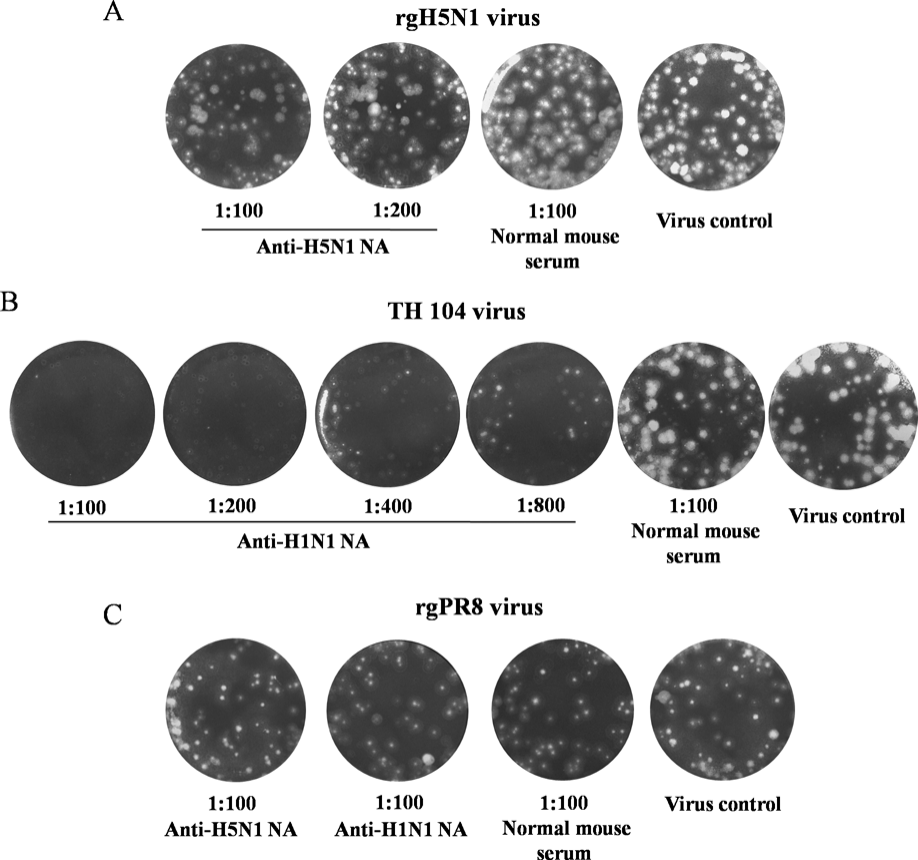
Inhibitory effect of anti-H5N1 NA and anti-H1N1 NA antisera determined by plaque reduction assay in MDCK cell monolayers. The results demonstrate the reduction in number of plaques and plaque size mediated by anti-H5N1 NA and anti-H1N1 NA antisera compared to normal mouse serum and virus controls. Pooled mouse sera collected at 6 weeks p.i. were employed in the assay.

To determine the inhibition of virus release, the MDCK cell monolayers were infected with the test virus prior to adding with the media containing anti-NA antisera at various dilutions. Using the sera at dilution of 1: 100 as the cut-off value, the result demonstrated that anti-H5N1 NA antisera reduced the number of plaques of rgH5N1 virus by 25%, and 80% of the plaques were at size of 1 mm compared to the cultures treated with normal mouse sera in which 80% of the plaques were at size of > 2 mm in average (Fig 8A). Anti-H1N1 NA antisera could inhibit the wild type TH 104 virus to the serum dilution of 1: 800 (Fig 8B). However, both kinds of immune sera at dilution of 1:100 could not inhibit plaque formation of rgPR8 virus (Fig 8C). We concluded that the replication inhibitory activity of anti-NA antisera varied according to virus strain.

### Inhibitory Effect of Anti-NA Antisera on Viral Replication

**Fig 9:**
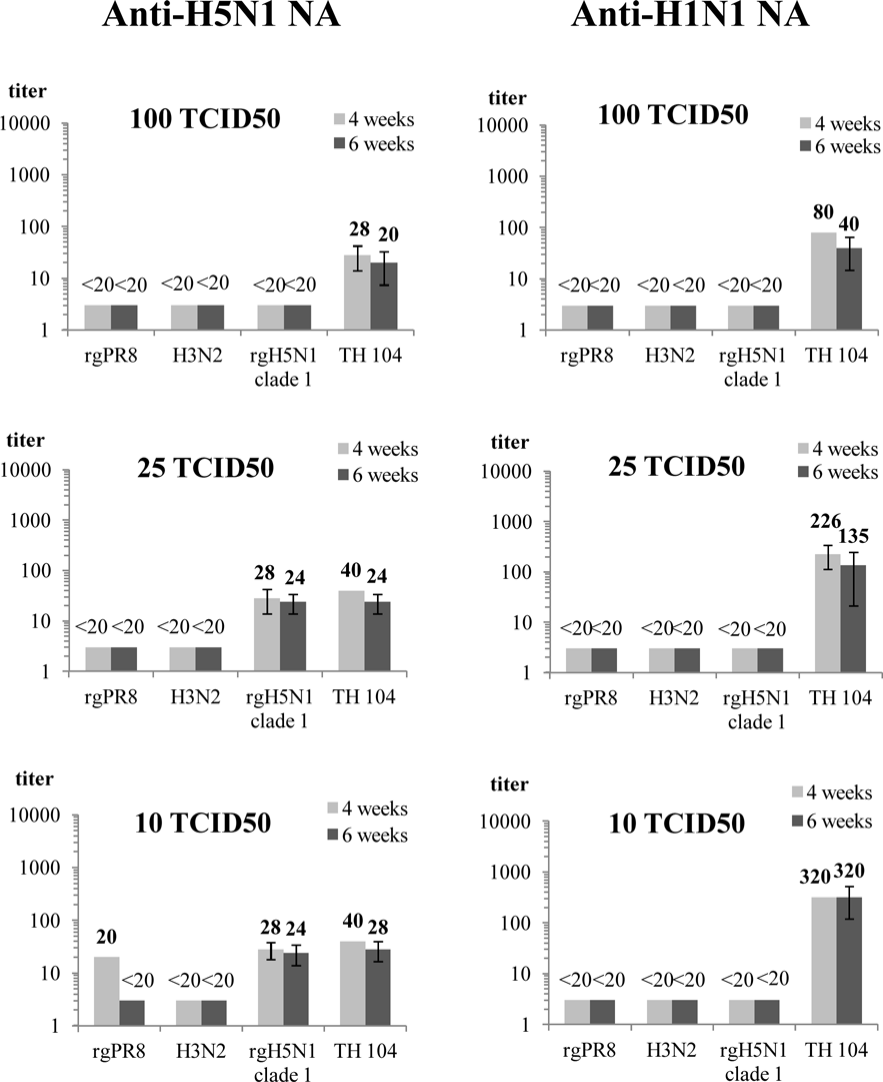
Inhibitory effect of anti-NA antisera against 3 virus concentrations (100, 25, and 10 TCID50) as determined by virus replication inhibition assay. Anti-H5N1 NA antisera modestly inhibit replication of reverse genetic (rg) virus carrying homologous NA and show moderate level of antibody titers that cross inhibit the H1N1pdm virus. On the other hand, anti-H1N1 NA antisera well inhibit replication of the H1N1pdm virus, but not cross inhibit rgH5N1 virus. Both kinds of anti-NA antisera do not inhibit rgPR8 virus and H3N2 virus. No significant difference in anti-NA antibody titers is found between pooled mouse sera collected at 4 weeks and 6 weeks p.i. (Mann-Whitney U test, *p* > 0.05). The geometric mean antibody titers are calculated. The error bars indicate standard deviations of 2 independent experiments for each lot of the test sera.

Mouse immune sera at a starting dilution of 1:20 was 2-fold serially diluted and assayed against various viruses at 3 test concentrations, 100, 25 and 10 TCID50. The result showed that antisera from mice immunized with rVac-H5N1 NA virus did not inhibit the replication of rgH5N1 virus at the concentration of 100 TCID50, but poorly inhibited TH 104 H1N1 virus at the same virus concentration. When concentrations of the test viruses were lowered to 25 and 10 TCID50, some degree of inhibition against both rgH5N1 and H1N1 viruses was observed. Sera from mice immunized with rVac-H1N1 NA virus exhibited inhibitory activity only on homologous H1N1 virus at all 3 test concentrations investigated, but no cross replication inhibition was observed for rgH5N1, rgPR8 and wild type H3N2 virus (Fig 9). The replication-inhibition antibody titers in mouse sera collected at 6 weeks p.i. were slightly lower than those collected at 4 weeks p.i. Nevertheless, the differences were not statistically significant (Mann-Whitney U test, *p* > 0.05). Concordant results were obtained from the two lots of mouse sera collected at 6 weeks p.i..

## Discussion

Herein, recombinant vaccinia viruses harboring *NA* gene insert derived from HPAI H5N1 virus or 2009 pandemic H1N1 virus were constructed and used as the immunizing agents to raise anti-NA antibodies in BALB/c mice. With these recombinant viruses, the NA protein was produced without interference from other influenza genes. These recombinant NA proteins were characterized for their biological and immunological properties. Similar to the previous reports [34, 43], we showed that NA proteins were localized in the cytoplasm and in particular on the cytoplasmic membrane of TK^−^ cells infected with recombinant vaccinia virus. However, NA proteins encoded by different virus strains and in different cell types had different MW as determined by WB assay. The TK^−^ cells infected with rVac-H1N1 NA virus displayed a band of NA protein at MW of 75 kDa; whereas MDCK cells infected with H1N1 wild type virus displayed two bands of NA proteins at MW of 75 and 55 kDa. After PNGase F digestion, only one band at MW of 50 kDa was obtained. This MW is close to 51.6 kDa, the size of NA protein determined from the amino-acid sequence using web-based tools. This finding suggested that H1N1 NA protein was hyperglycosylated by *N*-linked oligosaccharides. Nevertheless, the other study showed that inactivated A/California/7/2009 (H1N1) virus (Cal virus) or Vero cells infected with recombinant vaccinia virus Lister strain carrying NA derived from Cal virus or DF-1 chicken cells infected with recombinant modified vaccinia virus Ankara strain carrying *NA* gene derived from Cal virus expressed only one form of NA protein at MW of 75 kDa [19]. Our study also demonstrated that the NA protein synthesized in MDCK cells infected with HPAI H5N1 wild type virus or TK^−^ cells infected with rVac-H5N1 NA virus had the same MW of 55 kDa. The higher MW of H1N1 NA may be due to higher number of glycosylation sites as predicted by our previous study [40], and also the putative higher level of glycosylation compared to H5N1 NA as expected in this study. Hyperglycosylation of NA was also found in yeast expression system as reported for 3 virus subtypes from 3 independent studies, i.e., A/Vietnam/DT-036/2005(H5N1) [44], A/Victoria/3/75 (H3N2) [14] and A/new Caledonia/20/99(H1N1) [15]. Unfortunately, potential glycosylation site of N1 NA protein had not been determined in those studies. Our study also demonstrated that H5N1 NA (low level of glycosylation) had significantly higher sialidase enzymatic activity compared to H1N1 NA (high level of glycosylation) as determined either by MUNANA assay or ELLA. This result may be due to the hindrance of the glycosylated binding site on the NA globular head to bind the substrate. Nevertheless, the shorter length of NA stalk of H5N1 virus might also contribute to higher NA enzymatic activity as demonstrated by previous study [45]. Moreover, level of glycosylation has been previously shown to affect the magnitude of antibody response. Yang, et al demonstrated that the optimal level of glycosylation could elicit the higher antibody response; while hyperglycosylation or no glycosylation elicited the lower antibody titers [15].

Partial protection mediated by anti-NA antibodies had been suggested based on epidemiological data in human population as well as the study in animal models. Few *in vitro* studies have explored the mechanism of partial protection mediated by anti-NA antibodies. Ferrets immunized with the 2009 H1N1 pandemic virus developed anti-NA antibodies that cross inhibited HPAI H5N1 virus and *vice versa* as shown by NI assay and plaque size reduction assay [12]. Chicken sera with high NI antibody titers interfered with the binding of influenza virus to MDCK cells [46]. On the other hand, monoclonal antibodies originated from mice immunized with A/Brisbane/59/2007 (H1N1) virus provided protection to 80% of mice against the infection with A/California/07/2009 virus, even though the antisera from immunized mice did not inhibit the NA enzymatic activity of California virus [31]. These results suggested that anti-NA antibody may exhibit more than one mechanism of protection against influenza virus infection.

In the present study, antisera from mice immunized with rVac-H5N1 NA of clade 1 origin or rVac-H1N1 NA virus were assayed for their functional activities of anti-NA antibody by ELLA, plaque reduction assay and replication-inhibition assay in MDCK cell monolayers. Using ELLA, we showed that anti-H5N1 NA antibody blocked NA enzymatic activity of the rgH5N1 clade 1 and clade 2.3.4 viruses to the high antibody titers, but poorly cross inhibited the H1N1 NA. In contrast, anti-H1N1 NA antibody strongly inhibited both H1N1 NA and H5N1 NA. Nevertheless, both anti-H5N1 NA and anti-H1N1 NA antibody did not inhibit PR8 NA or H3N2 NA. Cross reactivity between anti-NA antibodies against H1N1 pandemic 2009 and H5N1 viruses were previously reported [12, 24, 47, 48]. Our analysis on the NA amino acid sequences of different viruses showed 86% identity of NA protein between 2009 H1N1pdm and PR8 viruses, 88% between H5N1 and PR8 viruses, and 91% for H1N1pdm and H5N1 viruses. Genetic analysis showed a close relationship between H1N1pdm NA and H5N1 NA such that both belonged to the same cluster (avian lineage) [49], whereas those NA of the H1N1 strains circulating before 2009 belonged to different clusters. On the other hand, NA from H3N2 strains belonged to different node in a phylogenetic tree (Fig 7). This study demonstrated that anti-NA antibody was subtype specific; and cross reactivity was observed with the viruses belonging to the closely related NA phylogenetic group only. It is to be further explored whether breadth of anti-NA antibody could be increased by booster immunization. It had been previously shown that chicken immunized with recombinant vesicular stomatitis virus carrying *NA* gene originated from A/chicken/Yamaguchi/7/04 (H5N1) virus and followed by one booster, exhibited high NI activity against homologous virus and to a lesser extent to the heterologous viruses of the same NA subtype, i.e., PR8 (H1N1) and A/New Caledonia/20/99 (H1N1) viruses [46].

Using plaque reduction assay, our study demonstrated that anti-NA antisera could reduce both the number of plaques and plaque size compared to the serum controls. However, the inhibitory activity of anti-NA antisera on plaque formation varied according to virus strain. We further investigated for the mechanism of action of NA antibody on virus entry and virus release using the test virus with homologous NA. Our result demonstrated that anti-NA antibody blocked the virus release, but not virus entry (data not shown). The blocking of influenza virus release by anti-NA antibody was well documented by various groups of investigators [12, 31–33, 46, 50]. However, the report on blocking of influenza virus entry by anti-NA antibody was scant [46].

The present study developed a method termed “replication-inhibition assay”, a simple *in vitro* technique used to demonstrate the inhibition of virus replication mediated by anti-NA antibody. Antibody targeting H1N1pdm NA inhibited the replication of H1N1pdm virus only, but not inhibited rgH5N1 viruses, rgPR8 virus and H3N2 viruses. Antibody to H5N1 NA elicited an inhibition against rgH5N1 viruses at virus concentrations of 25 and 10 TCID50, but not 100 TCID50; while cross inhibition against H1N1pdm virus was observed with all 3 virus concentrations. This finding suggested that anti-NA antibody might contribute only a modest replication-inhibition effect against highly virulent H5N1 virus. It was noted that mice immunized with rVac-H1N1 NA virus developed higher anti-NA antibody titer than those immunized with rVac-H5N1 NA virus, either determined by IFA, NI assay, plaque reduction assay or replication-inhibition assay.

Mouse sera collected at 6 weeks p.i. had a significantly higher NA inhibitory activity compared with those collected at 4 weeks p.i. as determined by ELLA, while no significant difference was observed by replication-inhibition assay. The measurement of NI antibody titers in term of the OD values made the NI assay more sensitive than the replication-inhibition assay. On the other hand, we could not exclude that the NI antibody and the replication-inhibiting antibody bind to different epitopes on the NA molecule, as it had been shown that monoclonal antibody without NI activity could protect mice against the lethal virus challenge [31].

Currently, the importance of NA antigen in influenza vaccines has been widely discussed. We demonstrated that NA protein could induce moderate level of antibody that might prevent influenza virus infection through various mechanisms. Although NA antibodies were detected after immunization with inactivated vaccine [51], the amount of NA antibody response varied by vaccine lot [52, 53]. Thus, the recommendation of a standard amount of NA protein for vaccine content should improve vaccine efficiency, and in particular, broaden the immune protective role of influenza vaccine against emerging influenza strains [54, 55].
